# Prevalence of Root Canal Treatment During Orthodontic Treatment: A Retrospective Five-Year Follow-Up

**DOI:** 10.7759/cureus.35392

**Published:** 2023-02-24

**Authors:** Abdulaziz S AbuMelha

**Affiliations:** 1 Department of Restorative Dental Sciences, King Khalid University College of Dentistry, Abha, SAU

**Keywords:** randomised clinical trial, resorption, endodontics, root canal treatment, orthodontic treatment

## Abstract

Background: Orthodontic and endodontic treatments are commonly studied. It has been advocated that orthodontic treatment can cause an impact on endodontically treated teeth, and root canal therapy (RCT) during orthodontic intervention can cause a halt in treatment, thus prolonging the treatment duration. The objective of the present study was to evaluate the incidence of RCT among patients subjected to orthodontic treatment.

Materials and methods: The present retrospective study was done on 814 patients undergoing orthodontic treatment. The clinical and radiographic records were assessed for all patients, and evidence of RCT was calculated. The data were obtained about gender, type of teeth involved, and diagnosis of the condition.

Results: Out of those 658 patients who underwent orthodontic treatment in the past five years, 89 (13.53%) had undergone RCT, with the mean age being 21.38 years. A statistically significant difference (p<0.05) was found between both genders concerning diagnosis. A maximum of 24.7% of cases subjected to RCT were right mandibular first molar, followed by left maxillary first molar, and left mandibular first molar, with a statistically insignificant difference (p>0.05) between both genders concerning the type of tooth involved.

Conclusion: A significant rate of prevalence of RCT was observed among the patients undergoing orthodontic treatment, mainly involving molars. Males showed more incidence of RCT of teeth among patients subjected to orthodontic treatment. Thus, patients need to be evaluated for RCT before initiating orthodontic treatment.

## Introduction

For decades, a link has been established between endodontic and orthodontic treatment [1]. During orthodontic treatment, microscopic root resorption is a discrete characteristic observed in all permanent teeth. It is clinically insignificant and radiologically invisible. It has been found that orthodontic tooth movement is not feasible without this microscopic resorption [2]. It has become routine to do orthodontic management in patients with endodontically treated teeth. This is because of an increased level of awareness as well as raised demand for perfect esthetics by patients of older age groups [3].

Various studies have advocated a relationship between the treatment planning phases of orthodontic and endodontic treatments. Most of the studies in the literature have been conducted with a key focus on the effect of tooth movement during orthodontic treatment upon the dental pulp vitality and the apical root resorption severity on an endodontically treated tooth [4]. On the other hand, some studies advocated that teeth treated with root canal treatment (RCT) during the orthodontic tooth movement show less susceptibility to root resorption at the apex [5,6]. These concepts have a direct impact on understanding the effect of orthodontic treatment on the pulp vitality of the tooth, thereby affecting the prognosis of endodontically managed teeth, mainly during the impact of orthodontic forces on that tooth [7].

To date, no substantial evidence has been reported to explain the impact of orthodontic factors on root resorption in endodontically treated teeth. The external root resorption, which occurs due to orthodontic forces, occurs in two ways: First, resorption observed on the tooth surface by the cemental loss; secondly, if the surface of resorption is the apical portion of the root, it is depicted as the tooth shortening or root blunting [8]. The resorption of the root can be classified as mild or clinically insignificant (<2 mm) and severe or clinically significant with >4 mm or >1/3rd of root length being resorbed.

There exist differences in view about endodontically and orthodontically treated teeth. Authors advocate the possibility of excessive root resorption due to the orthodontic forces exerted on the endodontically treated teeth compared to a vital tooth. Limited literature is available that reveals the incidence of endodontic treatment in teeth subjected to orthodontic intervention. The present retrospective study was conducted to assess the prevalence of endodontic or root canal treatment during an orthodontic intervention.

## Materials and methods

This retrospective study was conducted in the Department of Orthodontics, King Khalid University College of Dentistry, by assessing the record of the past five years, from 2016 to 2021. The institutional ethical committee approved the study (IRB/KKUCOD/ETH/2022-23/025).

Patient selection

All patients who had been reported and registered for orthodontic intervention between the ages of 18 and 30 years were included in the study. With the help of G*power software and based on calculations assessed from a previous study [8], the sample size of 800 was calculated. The permanent maxillary and mandibular teeth - canines, first and second premolars, and first molars - were assessed.

Inclusion and exclusion criteria

Inclusion Criteria

(i) Patients of any gender and above 18 years of age and (ii) patients undergoing orthodontic treatment were included.

Exclusion Criteria

(i) Patients with a history of extraction of teeth because of caries, traumatic injury, or previous orthodontic treatment and (ii) patients having congenitally missing premolars and molars were excluded.

Data collection

The data were collected from the hospital's electronic database. A complete patient record was assessed, including his/her case history, dental records, study models, and radiographic investigations. Each patient was assessed from his/her radiographic record of orthopantomograms (OPGs).

Data assessment

Once complete data were collected, every patient detail was recorded after assessing clinical and radiographic records. If any evidence of endodontically treated tooth or teeth was present, the records were cross-examined after correlating with the clinical data. The dental history and case sheet records were checked for any RCT. Once data were collected, it was analyzed with IBM SPSS Statistics Software Version 20.0 (IBM Corp, Armonk, NY). A thorough descriptive analysis was done to record the prevalence of RCT in patients who underwent orthodontic treatment. Frequency, percentage, and mean were calculated among both genders. A chi-square test was used to establish the association between diagnosis and RCT and the gender of the patient.

## Results

A total of 814 patients were assessed from the database, and out of those, 658 (80.84%) patients in the study underwent orthodontic treatment in the past five years. Of that, 46.5% were males and 53.49% were females (Table 1).

**Table 1:** Incidence of patients who had undergone RCT RCT: root canal therapy.

	Total patients	Included	Males	Females	Had RCT
Quantity (n)	814	658	306	352	89
Percentage	100	80.84	46.50	53.49	13.53

Out of 658 orthodontically treated patients, 89 (13.53%) had undergone RCT, out of which, 38 (42.7%) were males and 51 (57.3%) were females. Among both genders, most of the subjects were aged 20-30 years, with the mean age being 21 years (Table 2).

**Table 2:** Distribution of study subjects according to demographic variables

Demographic parameters		Male		Female		Total	
		Quantity	Percentage	Quantity	Percentage	Quantity	Percentage
Gender	Female	0	0	51	100	51	57.3
	Male	38	100	0	0	38	42.7
Age groups	<20 years	7	18.42	8	15.68	15	16.85
	20-30 years	31	81.58	43	84.31	74	83.15
Total		38	100	51	100	89	100

We also recorded the diagnosis of teeth subjected to RCT. Most of the teeth, 62.9%, were decayed, and 37.1% were diagnosed as necrosed. Chi-square statistical analysis revealed a statistically significant difference (p<0.05) between both the genders concerning diagnosis (Table 3).

**Table 3:** Distribution of study subjects according to diagnosis

Diagnosis	Male		Female		Total	
	Quantity	Percentage	Quantity	Percentage	Quantity	Percentage
Necrotic	14	36.8	19	37.3	33	37.1
Decayed	24	63.2	32	62.7	56	62.9
Total	38	100	51	100	89	100
Chi square	2.229					
p-Value	0.031					

On assessing the teeth being subjected to RCT, it was found that a maximum of 24.7% cases were of right mandibular first molar (#46), followed by 13.5% cases being of left maxillary first molar (#26), and 12.4% involving left mandibular first molar (#36), with a statistically insignificant difference (p>0.05) between both the genders concerning the type of tooth involved. It was also noticed that males suffered a higher incidence of RCT than females (Table 4).

**Table 4:** Distribution of study subjects according to root canal-treated teeth numbers

Tooth no.	Male		Female		Total	
	Quantity	Percentage	Quantity	Percentage	Quantity	Percentage
13	3	7.9	3	5.9	6	6.7
14	4	10.5	2	3.9	6	6.7
15	1	2.6	3	5.9	4	4.5
16	2	5.3	4	7.8	6	6.7
23	3	7.9	3	5.9	6	6.7
24	1	2.6	5	9.8	6	6.7
25	2	5.3	0	0	2	2.2
26	6	15.8	6	11.8	12	13.5
34	0	0	1	2.0	1	1.1
35	1	2.6	0	0	1	1.1
36	3	7.9	8	15.7	11	12.4
45	1	2.6	5	9.8	6	6.7
46	11	28.9	11	21.6	22	24.7
Total	38	100	51	100	89	100
Chi square	3.189					
p-Value	0.918					

## Discussion

In this study, we observed a considerable 13.53% prevalence rate of orthodontic patients subjected to endodontic treatment. Out of 658 orthodontic patients, 89 underwent RCT, mainly in molars. In accordance with our study, Verma and Pandian [8] reported a prevalence rate of endodontically treated posteriors being 8.7%. Similarly, Lupi-Pegurier L et al. [9] found a prevalence rate of 18.9%, which was higher than our study. This might be because of population variation.

We noticed that males suffered from a higher incidence of RCT than females. In contrast to our study, Lupi-Pegurier L et al. [9] found that males were less subjected to RCT than females. They observed that the maxillary first premolar was the tooth being the maximum treated endodontically, in contrast to our study as we reported that the incidence of RCT in molars was maximum. However, similar to our study, Verma and Pandian [8] found males to be significantly more affected than females, with the highest prevalence of RCT in the maxillary first molar.

Gulsahi K et al. [10] conducted a study on the Turkish population and reported a prevalence rate of root canal-filled teeth being 3.3% and significantly higher among females. This is in contrast to the results of our study, where males have undergone a significantly higher number of RCTs that too in molars. De Cleen et al. [11] conducted a study on the Dutch population and found that the prevalence rate of root canal-treated teeth was only 2.8%, which was relatively less than the results of our study. This might be due to the difference in the sample size, geographical area, and population studied based on age. They observed that mandibular first permanent molars showed a prevalence rate of 11.3%, which matched our study's results, where a higher prevalence rate was observed among mandibular first molars. Eriksen et al. [12] conducted a similar study on the Norwegian population and found a prevalence rate of 3.4% for root canal-treated teeth. Thus, various authors found that the rate of prevalence of RCT among patients undergoing orthodontic treatment ranges from 2% to 15%.

We can conclude that before starting the orthodontic intervention, the extent of caries should be adequately assessed. Improper pulp status can increase the chances of root resorption and infection, leading to a halt during the orthodontic intervention, thus increasing the treatment time [13]. When teeth get resorbed, infected, or fractured during an orthodontic intervention, the complete treatment plan is altered. As orthodontists, they must do an efficient treatment plan with the least dissonance to the teeth [14].

It has been observed that orthodontic treatment must not alter the state of root canal-treated teeth. Thus, it has been advocated that more physiological treatment mechanics should be involved in treating a patient with root canal-treated teeth. In most cases, the anterior can be retracted for a longer distance. For proper teeth and bone modeling, a sequential treatment plan should be followed [15].

This study has some limitations. The study was retrospective, so there might be some alterations in the rate of prevalence because of the presence of some incomplete records. The sample size was also limited. Thus, further prospective studies should be conducted over a long period using a large sample size.

## Conclusions

This study revealed a significant prevalence rate of RCT among patients undergoing orthodontic treatment. We observed that the most commonly involved teeth were molars, that too mandibular molars. Males showed more incidence of RCT of teeth among patients subjected to orthodontic treatment. Thus, patients need to be evaluated for the requirement of RCT before initiating orthodontic treatment.
